# The pyramiding of *QYr.cib-3AS* and *YrT14* enhances wheat resistance to stripe rust

**DOI:** 10.3389/fpls.2026.1802598

**Published:** 2026-04-22

**Authors:** Jianming Hu, Jiasheng Cheng, Yunfang Li, Fangpu Han, Lei Zhang, Yu Wu

**Affiliations:** 1 Chengdu Institute of Biology, Chinese Academy of Sciences, Chengdu, Sichuan, China; 2 University of Chinese Academy of Sciences, Beijing, China; 3 Institute of Food Crops, Yunnan Academy of Agricultural Sciences, Kunming, Yunnan, China; 4 State Key Laboratory of Plant Cell and Chromosome Engineering, Institute of Genetics and Developmental Biology, Chinese Academy of Sciences, Beijing, China

**Keywords:** *Triticum aestivum*, stripe rust, BSA-seq, QTL mapping, CAPS/dCAPS

## Abstract

Wheat stripe rust, caused by *Puccinia striiformis* f. sp. *tritici*, is a globally prevalent, wind-borne fungal disease and remains one of the most destructive threats to wheat production. By combining BSA-seq and QTL mapping using the wheat 120K SNP array, we identified two genomic regions associated with stripe rust resistance. Among them, a QTL on the short arm of chromosome 3A, *QYr.cib-3AS*, was consistently detected across environments, explaining 20.10%–25.21% of the phenotypic variance and showing LOD of 2.57–3.58. *QYr.cib-3AS* was delimited to an interval flanked by dCAPS-78 and dCAPS-83. Additive-effect analysis showed that RILs pyramiding *QYr.cib-3AS* with *YrT14* increased stripe rust resistance by 77.20% relative to RILs lacking both *QYr.cib-3AS* and *YrT14*, indicating that the pyramiding strategy had a significant impact on stripe rust resistance and underscoring its importance for high-yielding cultivars with durable resistance. The candidate interval exhibited high collinearity. Among the 48 high-confidence genes annotated in this region, an integrated analysis of transcriptome data, functional annotation, and sequence variation suggested that *TraesCS3A03G0118200* and *TraesCS3A03G0119800* are key candidate genes underlying *QYr.cib-3AS*. Collectively, these findings provide a foundation for marker-assisted breeding and future cloning and functional characterization of stripe rust resistance genes, which may help accelerate the development of elite stripe rust–resistant wheat cultivars, thereby improving wheat resistance to this destructive pathogen.

## 1 Introduction

Wheat is adapted to diverse climates and soil types and is one of the most widely cultivated cereals worldwide, contributing about 20% of the total protein and calories in human diets, thereby making it a major contributor to global food security (Nazim Ud Dowla et al., 2018; Sansaloni et al., 2020). Globally, wheat is harvested from approximately 220 million hectares, and its annual production exceeds 800 million tonnes (FAOSTAT, https://www.fao.org/faostat/en/). However, as the global population continues to increase, annual wheat production must rise by approximately 60% by 2050 to meet future demand (Wheat Initiative, https://www.wheatinitiative.org/). Wheat production is affected by multiple diseases, among which stripe rust (also known as yellow rust), caused by the fungal pathogen *Puccinia striiformis* f. sp. *tritici* (*Pst*), is one of the most destructive fungal diseases threatening global wheat production. Stripe rust occurs in more than 60 countries across nearly all continents, and major epidemics have been reported in China, the United States, and Australia (Wellings, 2011; Savary et al., 2019). In recent years, newly emerged, highly aggressive rust pathotypes have posed an increasing threat to food security (Tong et al., 2024).

Major strategies for controlling wheat stripe rust include fungicide application, quarantine and regulatory measures, and cultural control. Among these approaches, discovering, characterizing, and introgressing effective resistance genes into wheat to develop resistant cultivars is considered the most practical and sustainable strategy for controlling this disease, owing to the relatively low cost and minimal environmental impact (Figueroa et al., 2018; Chen, 2020). To date, 87 wheat stripe rust resistance genes have been officially catalogued (Sharma et al., 2024). However, not all of these genes have been successfully deployed in wheat breeding programs, and many are no longer effective against *Pst* pathotypes that emerged or became prevalent after 2000, underscoring the persistent challenge posed by the pathogen’s dynamic evolution (Chen et al., 2021). Most of the identified genes confer race-specific resistance, also referred to as all-stage resistance (ASR), which is often short-lived because it can be overcome by the emergence of new virulent pathotypes (Nazarov et al., 2020; Singh et al., 2022). In contrast, adult-plant resistance (APR) genes typically confer quantitative resistance that is more durable and broad-spectrum, and is often governed by multiple genes (Singh et al., 2016). In most studies, APR genes rarely act alone (Xiang et al., 2024). For example, the cultivar CW86 carries *Yr29*, *Yr30*, and two additional genes (Zeng et al., 2019), while XN3157 carries *Yr29*, *Yr78*, and *QYrxn.nwafu-2AL* (later designated *Yr86*) (Huang et al., 2023). When a line carries *Yr29* alone, it generally exhibits a near-susceptible to moderately resistant response. Nevertheless, higher levels of resistance can be achieved when *Yr29* is combined with other genes (Chen, 2005; Singh et al., 2011; Chen, 2013). For the currently prevalent *Pst* pathotypes, the individual effects of APR genes such as *Yr18*/*Lr34*/*Sr57* (Krattiger et al., 2009), *Yr29*/*Lr46*/*Sr58* (William et al., 2003), *Yr30*/*Lr27*/*Sr2* (Spielmeyer et al., 2003), and *Yr46*/*Lr67*/*Sr55* (Moore et al., 2015) are often modest. However, pyramiding these genes to achieve additive effects among loci can markedly enhance overall stripe rust resistance in wheat. Therefore, to facilitate pyramiding of multiple resistance genes and avoid overreliance on a single genetic background, it remains essential to continuously discover, characterize, and utilize diverse stripe rust-resistant germplasm, thereby expanding the resistance gene pool needed for effective and sustainable control of stripe rust.

Researchers have advocated screening and mining stripe rust resistance genes from diverse gene pools. Potential sources of resistance include not only common wheat cultivars and landraces, but also wild progenitor species with homologous genomes to bread wheat, as well as domesticated *Triticum* species and related *Aegilops* species with genomes closely related to that of bread wheat (Zhou et al., 2020b). These wild relatives harbor abundant disease resistance–associated variation and loci, providing important resources for improving wheat resistance, and numerous resistance genes have been successfully introgressed and effectively utilized in breeding programs (Reif et al., 2005). For example, *Yr9* (Schlegel and Korzun, 1997; Mago et al., 2005; Wang et al., 2025) and *Yr83* (Li et al., 2020) were derived from rye; *Yr17* (Helguera et al., 2003), *Yr37* (Marais et al., 2005a) and *Yr87* (Sharma et al., 2024) from *Aegilops*; *Yr50* (Liu et al., 2013) and *Yr69* (Hou et al., 2016) from *Thinopyrum*. In addition, *Yr15* (Peng et al., 2000), *Yr35* (Marais et al., 2005b) and *Yr84* (Klymiuk et al., 2022) have been reported in wild emmer wheat. Collectively, these wild relatives have greatly enriched and strengthened the wheat resistance gene pool, highlighting their substantial potential as valuable sources of resistance genes for wheat disease improvement.

In a previous study, JM22 was used as the recurrent parent and was crossed with the wheat–*Thinopyrum intermedium* addition line TAI-14, from which a homozygous translocation line WT78-10 (Zhongke78) was selected from the progeny. WT78–10 not only exhibits favorable agronomic performance but also shows high levels of stripe rust resistance at both the seedling and adult-plant stages, and it was confirmed to carry *YrT14* (Guo et al., 2023). Here, we crossed WT78–10 with the highly susceptible wheat Zhongkexiumai (ZKXM) to develop a RIL population. The population was genotyped using the wheat 120K SNP array to construct a high-density genetic linkage map, and BSA-seq was combined with QTL mapping to dissect the genetic basis of stripe rust resistance and to develop diagnostic markers for MAS, thereby providing strong support for improving wheat stripe rust resistance and cultivar development. In addition, transcriptome sequencing and exome-based variant analysis were used to identify a cysteine-rich receptor-like kinase (CRK) gene and an NRT1/PTR family (NPF) gene associated with stripe rust resistance as candidate genes, laying a solid foundation for subsequent fine mapping, map-based cloning, and functional characterization.

## 2 Materials and methods

### 2.1 Materials

WT78–10 carries a wheat–*Thinopyrum intermedium* long-segment translocation on chromosome 6A, with alien chromatin largely replacing the 6AL arm (Guo et al., 2023). Employing the single-seed descent method, a mapping population comprising 182 F_6_ RILs was constructed. Field trials were conducted at two distinct experimental sites—Xindu District (30.95°N, 104.04°E) in 2024 and 2025, and Maerkang City (31.91°N, 102.10°E) in 2025.

### 2.2 Phenotyping

Field management followed local agronomic practices. Each accession was cultivated in single-row plots (1 m in length, 0.25 m row spacing, with one replication). To ensure uniform disease pressure, the highly susceptible line Chuanyu 12 was interspersed every ten rows. The currently prevalent *Pst* races (CYR34, CYR33, SU11, Hybrid46 and G22) were mixed in specific proportions to inoculate seedlings in the field, using Chuanyu 12 and Mingxian 169 as sources of inoculum. Disease assessments were conducted 18 to 25 days post-anthesis, at which point infection levels on the susceptible check Chuanyu 12 reached 90 to 100%. Stripe rust responses were recorded using a 0 to 9 infection type (IT) scale. The highest IT score observed per accession in each environment was used for downstream analyses. Best Linear Unbiased Predictions (BLUPs) were computed in R v4.4.1 (lme4 package), with genotype and environment treated as random effects. Broad-sense heritability (*H*^2^) was estimated as:


$$H^{2}=\frac{\sigma_{G}^{2}}{\sigma_{G}^{2}+\frac{\sigma_{GE}^{2}}{n_{env}}+\frac{\sigma_{e}^{2}}{n_{env}}}$$

where $\sigma_{G}^{2}$ represents genotypic variance, $\sigma_{GE}^{2}$ indicates the genotype-by-environment interaction variance, $\sigma_{e}^{2}$ denotes the residual variance, and $n_{env}$ is the number of environments.

### 2.3 Genotyping

Two parents and 70 RILs were genotyped by Tcuni Bioscience Inc. (Chengdu, China) using the 120K-4HWA wheat SNP array. To ensure data quality, BCFtools v1.22 (Danecek et al., 2021) was used to retain biallelic variants. VCFtools v0.1.16 (Danecek et al., 2011) was employed for quality-control filtering, retaining markers with minor allele frequency (MAF) ≥ 30% and a missing rate ≤ 20%. PLINK v1.9.0-b.7.7 (Purcell et al., 2007) was used to convert genotypes into a 0/1/2 matrix and then to retain SNPs polymorphic between the parental lines for subsequent analyses. Genomic variants were annotated using SnpEff v5.2 (Cingolani et al., 2012).

### 2.4 Bulk segregant analysis

Two bulks were constructed, each consisting of 30 RILs from the population. Bulked segregant analysis (BSA) was then performed using EasyQTLseqr v1.2.0. SNP index values were calculated with ZKXM as the reference parent. Default parameters were applied for quality control of the sequencing data, including filtering based on genotype quality (GQ ≥ 30) and allele depth (AD ≥ 6). The average SNP index within sliding windows was calculated using a window size of 2 Mb and a step size of 200 kb. Based on the population type and population size, 10,000 simulations were conducted for each bulk, and confidence intervals were estimated using the simulated quantiles (95% and 99%). The ΔSNP index was calculated as ΔSNP index = SNP index (S pool) - SNP index (R pool), where SNP index (S pool) represents the frequency of the ZKXM genotype in the susceptible bulk, and SNP index (R pool) represents the frequency of the ZKXM genotype in the resistant bulk.

### 2.5 Genetic map construction, linkage and collinearity analysis

Recombination maps were converted into a skeleton bin map using SNPbinner2 (Gonda et al., 2019) with parameters -r 0.01 and --min-bin-size 5000. Construction of the genetic linkage map (WT78–10 coded as a, ZKXM coded as b) was performed using JoinMap v5.0 (Van Ooijen, 2019). Assignment of linkage groups was achieved by aligning the flanking sequences of SNP markers to the IWGSC RefSeq v2.1 reference genome (CS v2.1) to determine their corresponding chromosomes and physical positions (Alaux et al., 2024). Markers with similarity ≥ 0.95 were considered redundant and removed. Marker ordering was determined using the regression mapping algorithm with default parameters, and recombination frequencies were converted into genetic distances using the Kosambi mapping function. Genetic linkage maps and the collinearity between the linkage maps and CS v2.1 were visualized using R/qtl v1.70 (Broman et al., 2003) and JCVI v1.5.5.dev1 (Tang et al., 2024), respectively. For the synteny analysis of the *QYr.cib-3AS* region, sequences were first aligned using minimap2 (Li, 2018), and syntenic relationships and structural variations were then identified with SyRI (Goel et al., 2019) and finally visualized by plotsr (Goel and Schneeberger, 2022).

QTL mapping was conducted in R v3.6.1 using the package qtl, taking into account the cross type and generation number. An initial genome-wide scan using Haley–Knott regression was performed to identify cofactors for subsequent analysis. A second scan using the multiple-QTL model (MQM) (Arends et al., 2010) was then conducted to detect QTLs at a significance level of 0.05 while accounting for the identified cofactors. QTLs with LOD ≥ 2.5 and an average phenotypic variance explained (PVE) ≥ 10% were defined as major-effect QTLs, whereas QTLs detected consistently across three environments were defined as stable QTLs. Regional scans were performed using MapQTL6 (Van Ooijen, 2009), and the results were plotted by Mapchart v2.32 (Voorips, 2002). QTL nomenclature followed the wheat genetic naming conventions (Boden et al., 2023), in which stripe rust resistance and the Chengdu Institute of Biology, Chinese Academy of Sciences, were abbreviated as “*Yr*” and “cib”, respectively.

### 2.6 Molecular marker development and polymorphic analysis

Based on the initially mapped interval, CAPS/dCAPS markers were designed using SNP Primer Pipeline2 (https://github.com/pinbo/snp_primer_pipeline2), and genotyping was performed on 182 progeny individuals. *YrT14* was detected using the previously reported specific primer YrT14K50 (Guo et al., 2023). PCR reactions were carried out on a Bio-Rad CFX96 thermal cycler in a 10 µL reaction volume containing 1.0 µL genomic DNA (100 ng), 5 µL 2X SanTaq PCR Mix (Sangon Biotech, Shanghai, China), 0.4 µL of each primer (10 µM), and ddH_2_O to adjust the final volume. The PCR amplification program consisted of an initial denaturation at 95 °C for 4 min, followed by 35 cycles of 95 °C for 20 s and 58–61 °C for 30 s, a final extension at 72 °C for 10 min, and a hold at 4 °C. Restriction enzyme reactions were performed in a 30 µL volume containing 10 µl of PCR product, 2 µL of NEBuffer, 10 U of restriction enzyme, and 17 µl of ddH_2_O. The reaction mixtures were incubated at 37 °C for 3 h, then analyzed on 2.5% agarose gel.

### 2.7 RNA−seq analysis and whole exome sequencing

RNA sequencing was performed on leaf tissues collected from field-grown WT78–10 and ZKXM at the adult stage, with three biological replicates per sample. To ensure high-quality short reads, paired-end sequencing reads were subjected to quality control using fastp v0.23.4 (Chen, 2025) with default parameters. To thoroughly remove reads originating from the wheat host, quality-controlled reads were classified using Kraken2 (Wood et al., 2019), and the wheat-assigned reads were mapped to the complete reference genome of CS v2.1 using STAR v2.7.11b (Dobin et al., 2013). Read counts were quantified with RSEM (Li and Dewey, 2011) and normalized to transcripts per million (TPM). Differential expression analysis was performed using DESeq2 v1.46.0 (Love et al., 2014). Genes with |log2 fold change| ≥ 1 and an adjusted *p* value< 0.05 were considered differentially expressed (DEG). Gene ontology (GO) and KEGG enrichment analyses were carried out using the DAVID database (Sherman et al., 2022).

WT78–10 and ZKXM were subjected to whole-exome sequencing (WES) on the DNBSEQ–T7 platform (~ 65.79× coverage). Sequencing reads were also aligned to the CS v2.1 using BWA-MEM (Vasimuddin et al., 2019). SNPs and Indels were then called using GATK HaplotypeCaller v4.1 (McKenna et al., 2010; Depristo et al., 2011). Low-quality SNPs were filtered out using the following criteria: QD< 2.0, MQ< 40.0, FS > 60.0, SOR > 3.0, MQRankSum< − 12.5, ReadPosRankSum< − 8.0; and low-quality indels were filtered: QD< 2.0, FS > 200.0, SOR > 10.0, MQRankSum< − 12.5, ReadPosRankSum< − 8.0.

### 2.8 Statistical analysis

Statistical analysis and graphing were performed using R’s built-in functions and multiple R packages, including tidyverse v1.3.2, ggplot2 v3.4.0, ggsignif v0.6.4, and ggpubr v0.5.0. Comparisons of expression levels or phenotypic differences between two groups were conducted using two-tailed Student’s *t*-tests. Comparisons of phenotypic differences among multiple groups were performed using one-way ANOVA, followed by Tukey’s HSD *post hoc* tests.

## 3 Results

### 3.1 Phenotypic variation in stripe rust resistance

**Figure 1:**
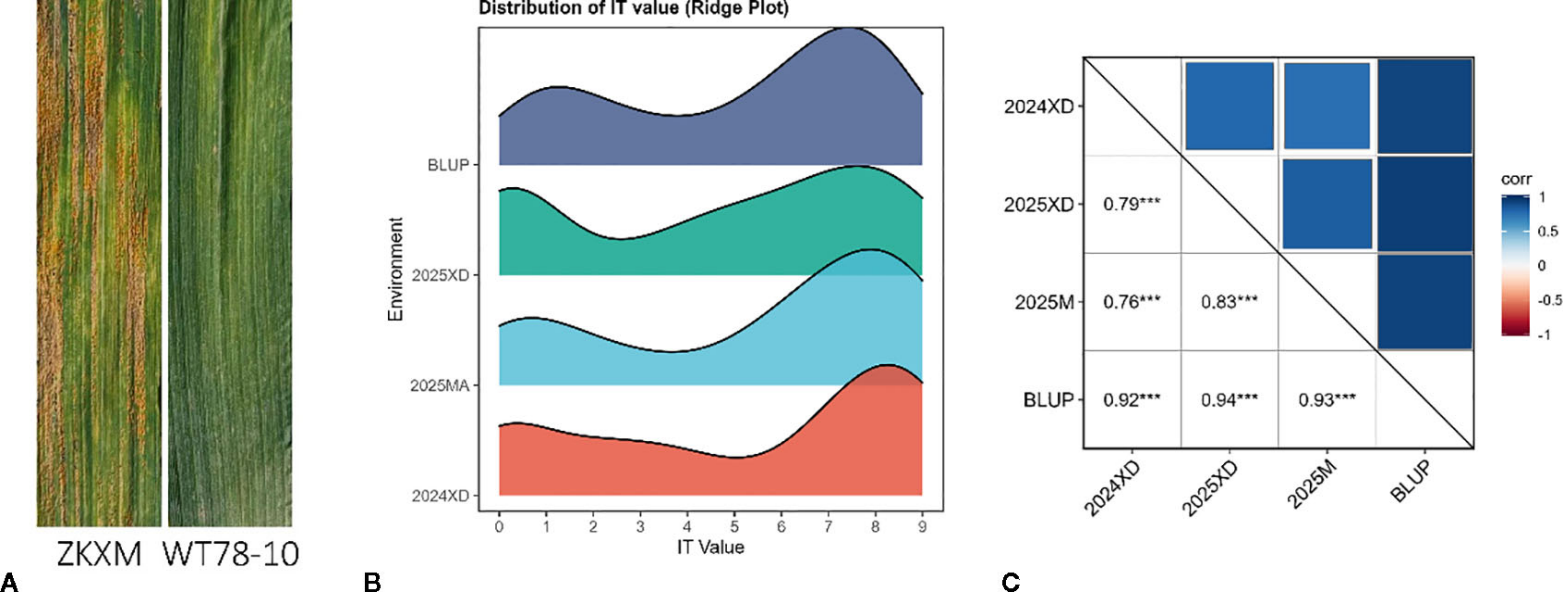
Stripe rust responses of the parents and phenotypic variation in the RIL population. **(A)** Stripe rust responses of ZKXM and WT78–10 in the field. **(B)** Frequency distribution of stripe rust IT in the RIL population across different environments. **(C)** Pearson’s correlation for stripe rust phenotypes across environments.

The RIL F_6_ population derived from a cross between WT78–10 and the highly susceptible wheat cultivar ZKXM displayed extensive and significant variation among progeny (**Figures 1A, B**). Across different environments, the Pearson correlation coefficients ranged from 0.76 to 0.83, and the broad-sense heritability was calculated as 0.92 (**Supplementary Table 1**), suggesting that stripe rust resistance within this germplasm population is stable across environments (**Figure 1C**). It can be inferred that the pathogen isolates may exhibit a certain degree of stability, stripe rust resistance is mainly controlled by genetic factors, and the same major-effect gene contributes to resistance. The IT of the progeny population showed a continuous bimodal distribution across different environments, including the BLUP, indicating that resistance in this population is likely conferred by a single major-effect locus.

### 3.2 Bulked segregant analysis identified the resistance gene

**Figure 2:**
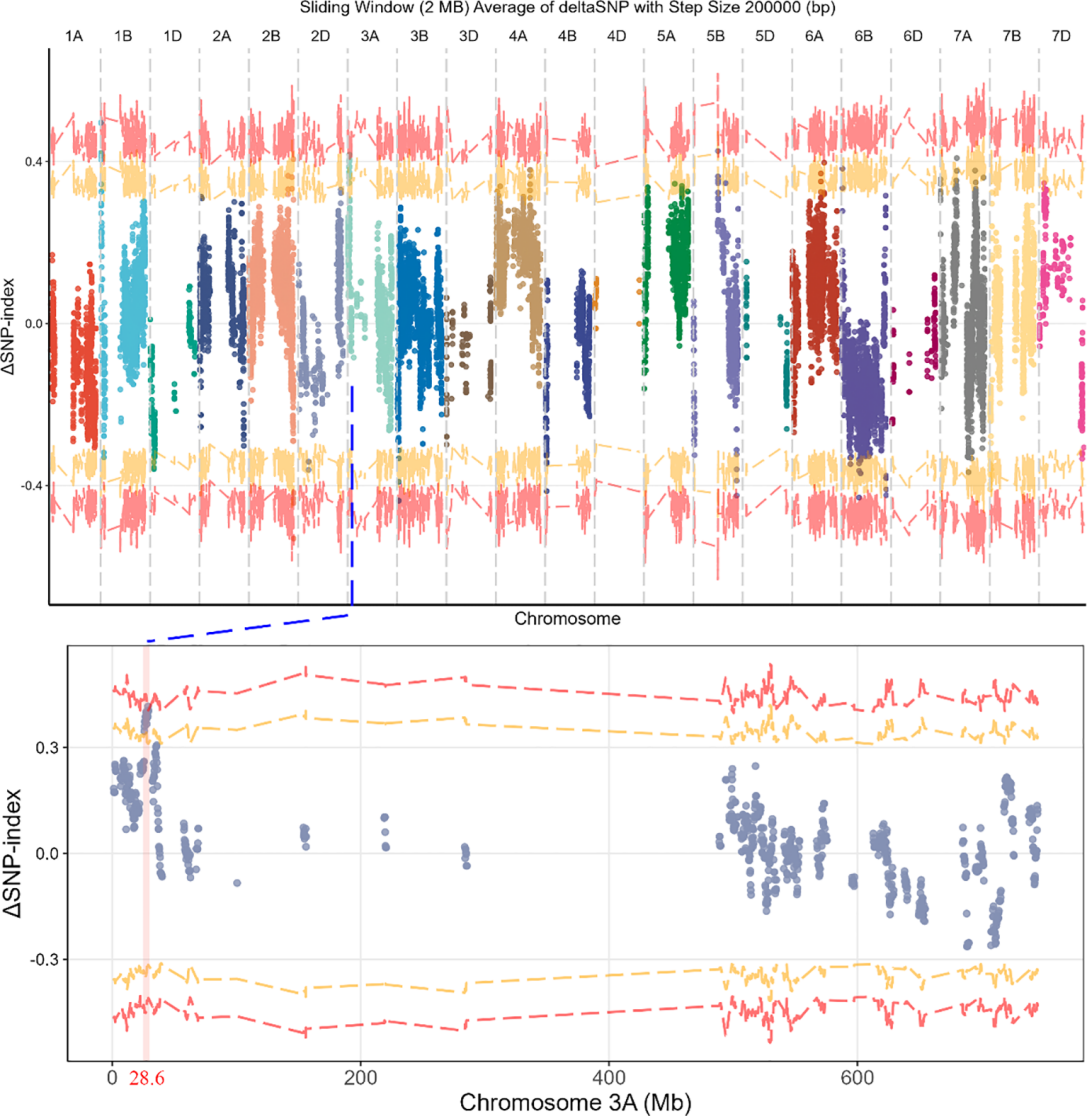
Whole genome and chromosome 3A ΔSNP-index.

After quality control, a total of 612,529 variants were retained for calculating the ΔSNP index between the bulks. Using a 95% confidence interval (CI95) threshold, 17 significant regions were detected across 12 chromosomes, including 1B, 1D, 2B, 3A, 3B, 4A, 4B, 5A, 5B, 6B, 7A, and 7B (**Supplementary Table 2**). Under a more stringent 99% confidence interval (CI99), only the region spanning 27.6–29.6 Mb on Chr 3A remained significant, with a positive ΔSNP index. This indicates that the frequency of the ZKXM allele is higher in the susceptible bulk than in the resistant bulk, suggesting that the resistance allele is derived from WT78-10 (**Figure 2**).

### 3.3 Genetic map construction and QTL mapping

A total of 685,042 variants were detected using the 120K wheat SNP array, of which 78,405 were polymorphic between the two parents. Based on these polymorphic loci, 1,614 recombination bins were constructed using 70 progeny individuals. After removing redundant markers, 578 bin markers were ultimately retained for linkage map construction (**Supplementary Figures S1A, B**). The resulting genetic map spanned a total genetic distance of 2,125.61 cM (**Table 1**). Collinearity analysis with the reference genome indicated no obvious large-scale chromosomal structural variations, and most markers were located toward the distal ends of chromosome arms, whereas relatively few markers were anchored in the pericentromeric regions (**Supplementary Figures S1C, D**). The map lengths for the A, B, and D subgenomes were 649.97, 796.77, and 678.87 cM, respectively, with average marker intervals of 3.33, 3.96, 3.73 cM per marker. The average marker interval across the entire linkage map was 3.68 cM. Overall, the constructed genetic map provides sufficient marker density for preliminary QTL mapping.

**Table 1:** Numbers and density of molecular markers on different chromosomes.

Chr	Mapped maker	Polymorphism maker	Bin maker	Non-redundant maker	Length (cM)	Average distance (cM)	Max spacing (cM)
1A	32918	3682	83	34	91.20	2.68	5.80
2A	40576	3769	67	26	91.31	3.51	10.80
3A	29657	3645	111	36	113.90	3.16	7.00
4A	34038	5434	81	28	95.75	3.42	8.60
5A	34151	4007	90	28	105.93	3.78	8.90
6A	29425	2931	56	13	38.30	2.95	9.40
7A	38513	5501	88	30	113.59	3.79	8.10
A Subgenome	239278	28969	576	195	649.97	3.33	10.80
1B	45812	5216	73	31	129.54	4.18	15.10
2B	48606	8388	82	27	95.68	3.54	10.20
3B	44496	6680	89	38	155.14	4.08	9.60
4B	24137	3077	63	24	89.23	3.72	9.10
5B	41413	2169	65	27	92.93	3.44	11.10
6B	40790	5782	59	24	85.45	3.56	10.00
7B	40579	4878	95	30	148.80	4.96	27.90
B Subgenome	285833	36190	526	201	796.77	3.96	27.90
1D	24248	2393	70	31	114.34	3.69	8.00
2D	32405	2986	73	27	90.47	3.35	10.30
3D	22314	2505	87	33	125.93	3.82	9.10
4D	12130	663	57	22	68.08	3.09	6.60
5D	27551	1563	65	22	83.31	3.79	6.70
6D	16984	1224	74	25	103.70	4.15	10.20
7D	24299	1912	86	22	93.05	4.23	6.90
D Subgenome	159931	13246	512	182	678.87	3.73	10.30
Whole Genome	685042	78405	1614	578	2125.61	3.68	27.90

**Figure 3:**
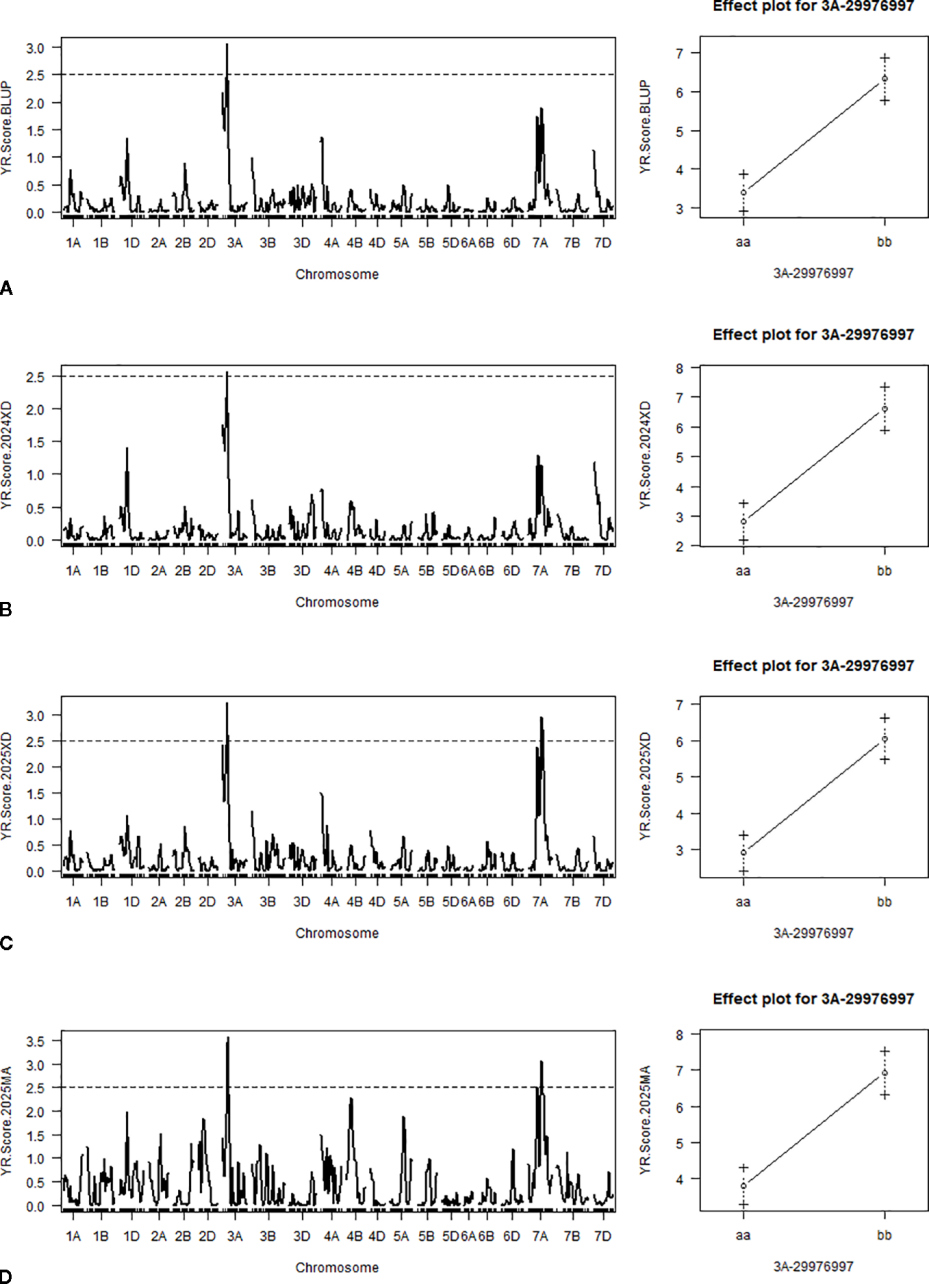
QTL mapping and additive effects for *QYr.cib-3AS*. **(A)** QTL mapping based on the BLUP dataset and additive effect analysis of *QYr.cib-3AS*. **(B)** QTL mapping in the 2024XD environment and additive effect analysis of *QYr.cib-3AS*. **(C)** QTL mapping in the 2025XD environment and additive effect analysis of *QYr.cib-3AS*. **(D)** QTL mapping in the 2025MA environment and additive effect analysis of *QYr.cib-3AS*.

Two QTLs were detected: one on chromosome 3A (*QYr.cib-3AS*) and one on 7A (*QYr.cib-7AL*). *QYr.cib-3AS*, located between markers 3A-26431044 and 3A-37517932, was consistently detected across all environments, explaining 20.10%–25.21% of the PVE (**Supplementary Table 3**). *QYr.cib-7AL* was identified in two environments, with the LOD peak around 65 cM, flanked by markers 7A-543126780 and 7A-561671119. Additive effect analysis indicated that resistance at both the *QYr.cib-3AS* and *QYr.cib-7AL* loci was contributed by WT78-10 (**Figure 3**; **Supplementary Figure S2**). The *QYr.cib-3AS* signal likely corresponds to the same locus identified by BSA-seq on chromosome 3A. By contrast, because the mapped interval for *QYr.cib-7AL* remains relatively large and its effect was detected only under some environments and conditions (including CI95 confidence peaks), we will increase the population size and further saturate this region with denser markers in future studies. Therefore, *QYr.cib-7AL* is not discussed in detail in the present study.

### 3.4 Mapping of the *QYr.cib-3AS* locus

**Figure 4:**
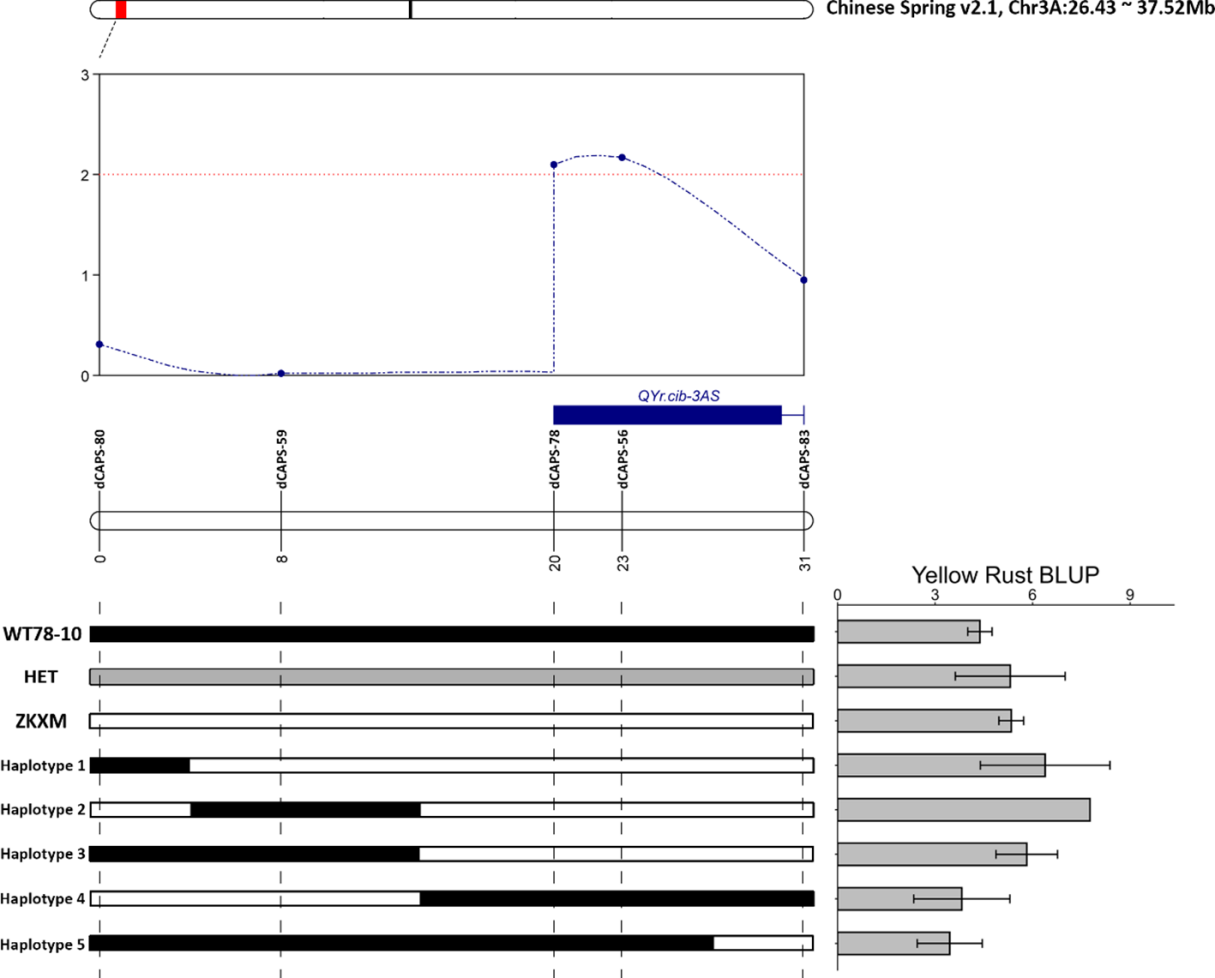
Regional mapping of *QYr.cib-3AS*.

Marker saturation and QTL mapping were conducted using 182 F_6_ RIL individuals. A total of 61 CAPS/dCAPS markers were developed within the candidate region on chromosome 3A, among which five markers showed stable polymorphism between the two parents. Based on the recombination events identified, *QYr.cib-3AS* was delimited to an approximately 11.0 cM interval between the markers dCAPS-78 and dCAPS-83 on the short arm of chromosome 3A (**Supplementary Table 4**). The physical interval was further narrowed to a 5.26 Mb region spanning 29.07–34.33 Mb on chromosome 3A (**Figure 4**).

### 3.5 Additive effects of the QTL

**Figure 5:**
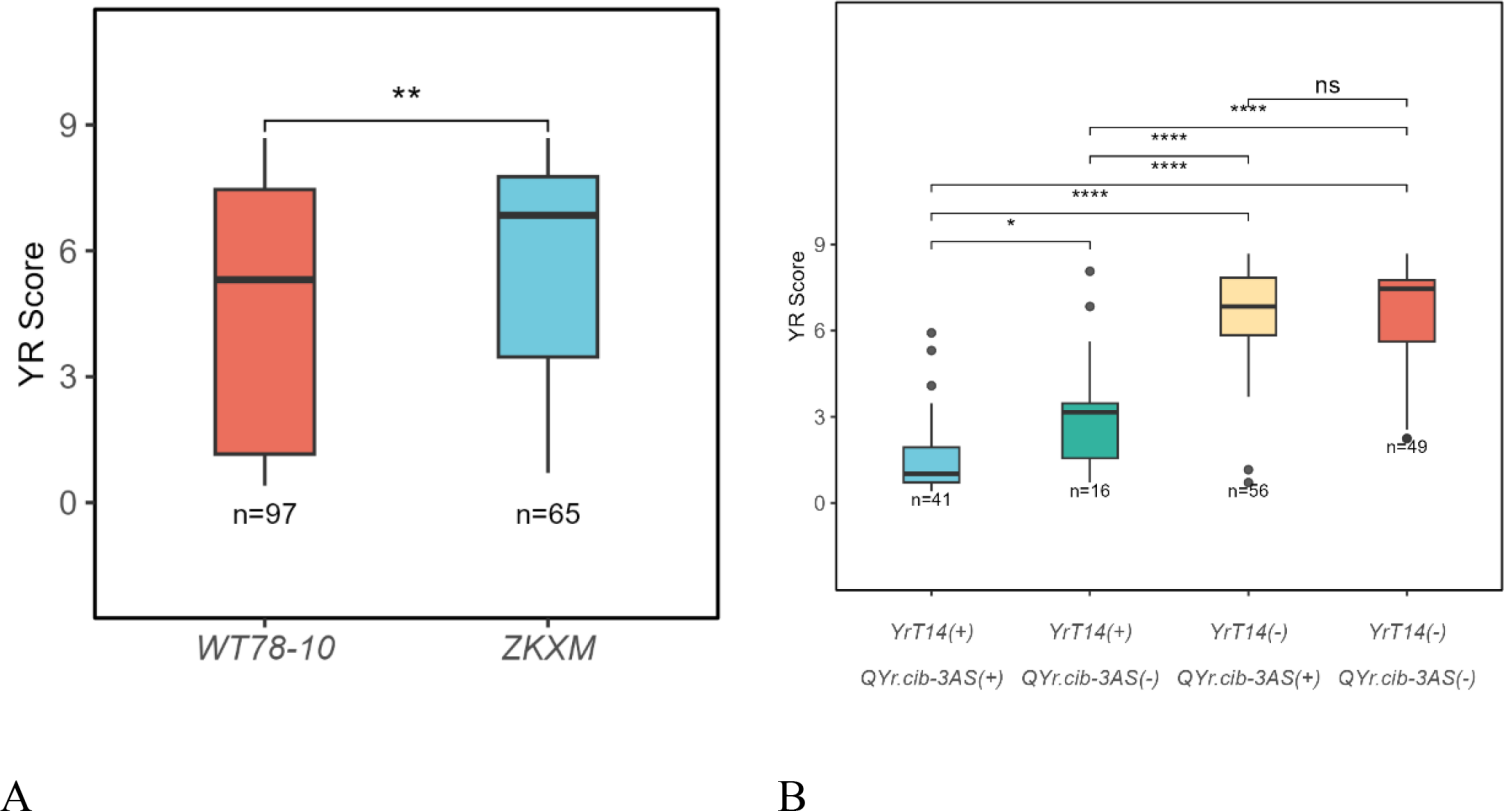
Additive effect analysis of *QYr.cib-3AS* and the 6A wheat–*Th. intermedium* translocation. **(A)** Additive effects of *QYr.cib-3AS*. **(B)** Additive effects of *QYr.cib-3AS* and *YrT14*. The asterisks indicate levels of statistical significance: * P < 0.05, ** P < 0.01 and **** P < 0.0001.

To evaluate the resistance effects conferred by *QYr.cib-3AS* and the 6A wheat-*Thinopyrum intermedium* translocation (*YrT14*) in combination, wheat lines were classified into different groups according to the genotypes of diagnostic markers. Lines carrying *QYr.cib-3AS* (+) and *YrT14* (+) showed mean infection type (IT) values of 4.49 and 2.14, respectively, whereas lines lacking these loci, *QYr.cib-3AS* (−) and *YrT14* (−), exhibited mean IT values of 5.85 and 6.75 (**Figure 5A**; **Supplementary Figure S3**). Notably, under severe stripe rust pressure, lines simultaneously carrying both loci displayed a markedly reduced mean IT value of 1.53, representing a 77.20% increase in resistance compared with lines lacking both loci (**Figure 5B**). Further analysis revealed a significant epistatic interaction between *QYr.cib-3AS* and *YrT14* (**Supplementary Table 5**). With *YrT14* acting as the core resistance locus, pyramiding it with *QYr.cib-3AS* is expected to facilitate the development of wheat cultivars with durable resistance to stripe rust.

### 3.6 Transcriptome analysis

After removing adapter sequences and low-quality reads, a total of 487.62 million reads were generated from the six samples. The average sequencing depths of the resistant and susceptible samples were 55.85× and 50.81×, respectively. The mean Q20, Q30, and GC contents across the samples were 97.42%, 93.02%, and 51.69% (**Supplementary Table 6**), respectively, indicating that the transcriptome sequencing data were of high quality.

**Figure 6:**
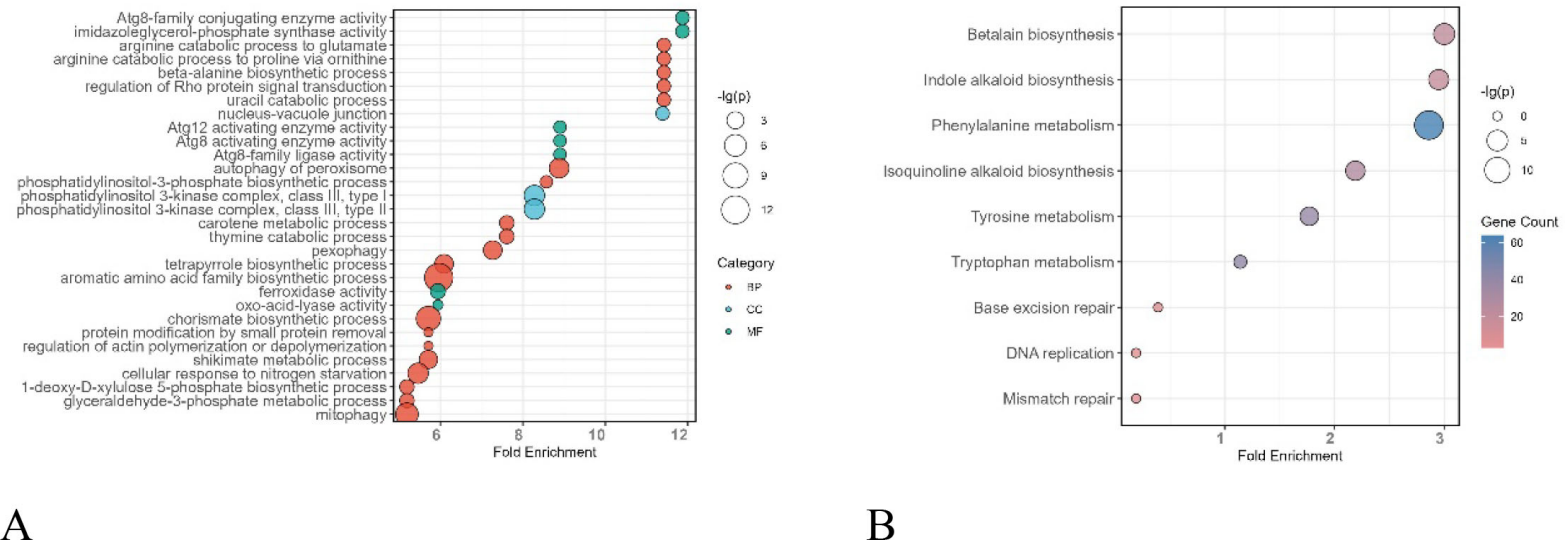
GO and KEGG. **(A)** GO enrichment analysis. **(B)** KEGG enrichment analysis.

In total, 10,580 DEGs were identified. GO enrichment analysis showed that these DEGs were mainly enriched in biological process (BP) terms, including amino acid catabolism and derivative biosynthesis, nucleotide metabolism, regulation of Rho/ROP protein–mediated signal transduction, and Atg8-related autophagy. These results suggest that stripe rust infection may induce metabolic pathways in wheat and activate cellular signaling regulation and autophagy, thereby enhancing cellular homeostasis and adaptive defense responses (**Figure 6A**). KEGG pathway enrichment analysis further revealed significant enrichment of genes involved in secondary metabolism and amino acid derivative biosynthesis, including phenylalanine metabolism, betaine biosynthesis, and indole alkaloid biosynthesis (**Figure 6B**). These pathways are closely associated with the biosynthesis of defense- and stress-responsive secondary metabolites, indicating that under stripe rust infection, wheat may enhance its adaptive defense responses by modulating amino acid metabolic pathways and promoting the activation of secondary metabolic processes.

### 3.7 Collinearity analysis

**Figure 7:**
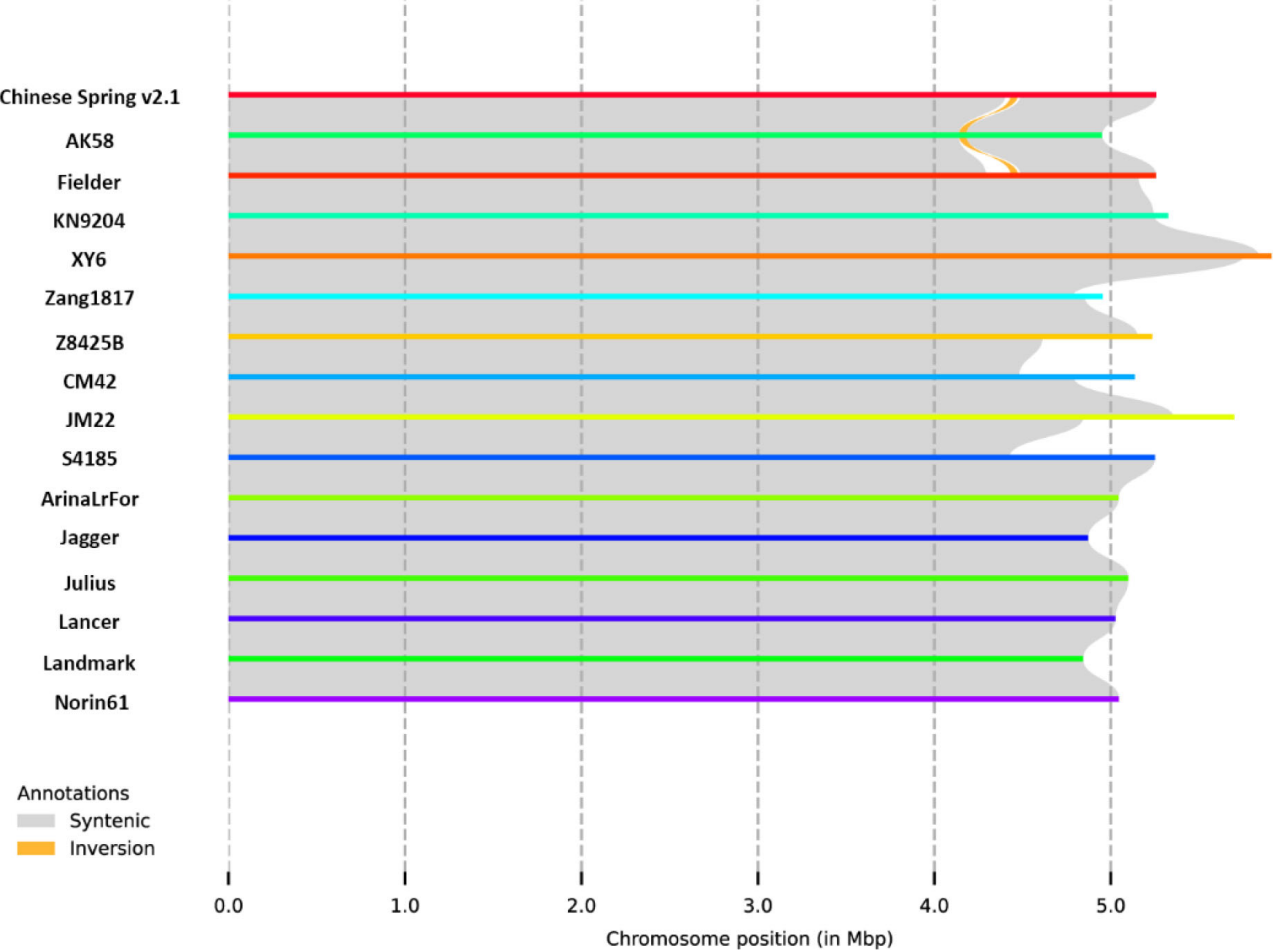
Collinearity analysis of the *QYr.cib-3AS* region.

A collinearity analysis of the *QYr.cib-3AS* region (29,073,232–34,330,582 bp) was conducted across multiple 16 chromosome-level wheat genome assemblies, including CS RefSeq v2.1, AK58, Fielder, KN9204, XY6, Zang1817, Z8425B, CM42, JM22, S4185, and 10+ wheat pangenome assemblies. The results showed that this region exhibits high collinearity among different genomes, with few large-scale insertions, deletions, or rearrangements detected (**Figure 7**). These findings indicate that the *QYr.cib-3AS* region is structurally highly conserved in wheat, and its genetic effects are more likely attributable to local sequence variation or gene regulatory differences rather than large chromosomal structural variations.

### 3.8 Gene expression profiles of candidate genes and sequence variation analysis

**Figure 8:**
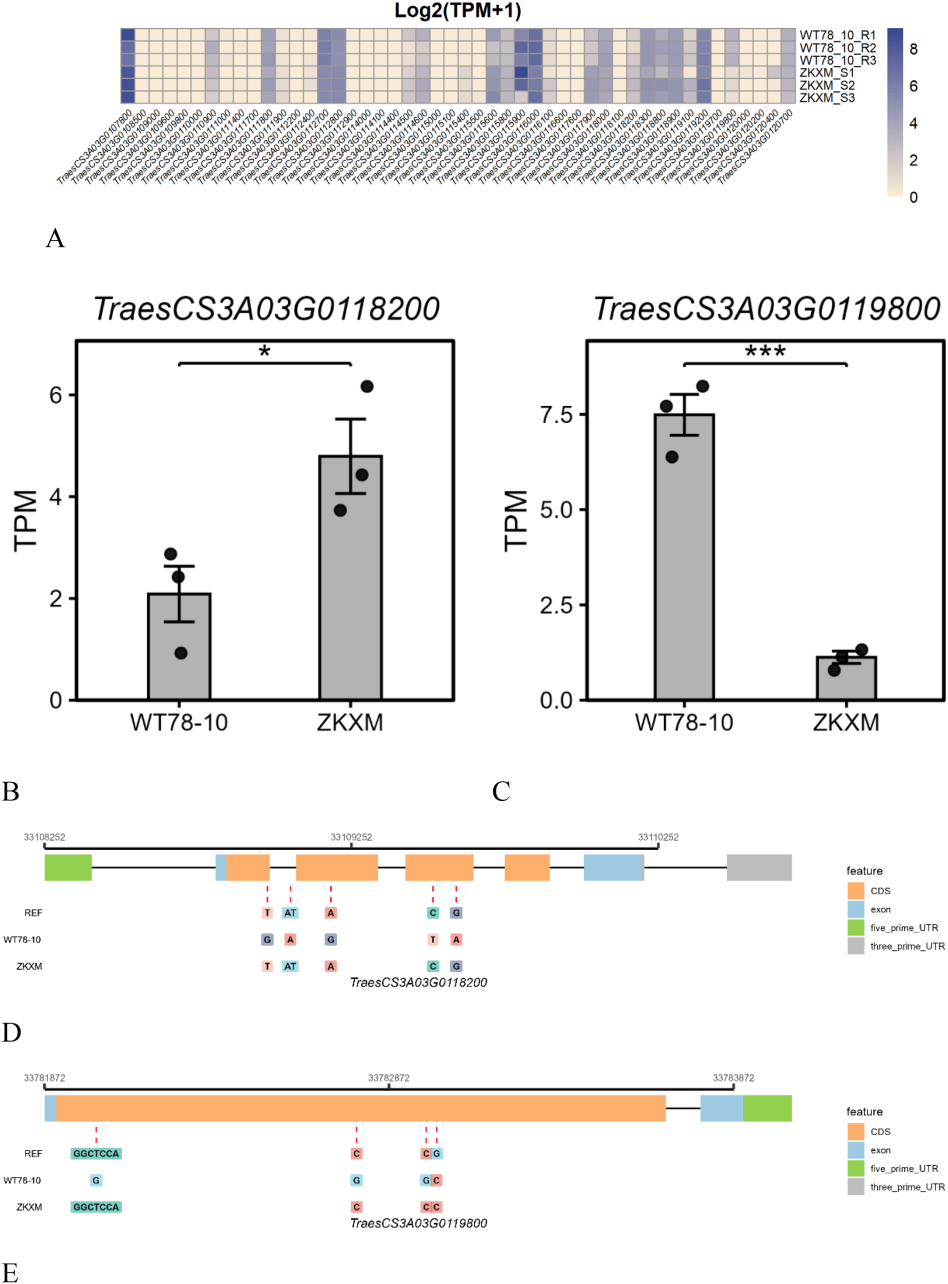
Gene expression profiles and candidate gene sequence variation. **(A)** Gene expression profiles. **(B, C)** Differential expression of *TraesCS3A03G0118200* and *TraesCS3A03G0119800* between the two parents at the adult-plant stage. **(D, E)** Sequence variation in *TraesCS3A03G0118200* and *TraesCS3A03G0119800* between the two parents. The asterisks indicate levels of statistical significance: * P < 0.05 and *** P < 0.001.

A total of 48 high-confidence genes were annotated within the candidate interval (**Figure 8A**). RNA-seq analysis of leaf tissues at the adult-plant stage showed that 22 genes were expressed (TPM > 1), and four genes exhibited a significant (*p* ≤ 0.05) expression difference between the two parents (**Figures 8B, C**). WES further revealed pronounced polymorphisms in *TraesCS3A03G0118200* and *TraesCS3A03G0119800* between the two parents. Five polymorphic sites were identified in *TraesCS3A03G0118200* (**Figure 8D**), including three synonymous variants (3A: 33108978, 3A: 33109185, 3A: 33109594), 1-bp deletion in an intron, and one missense variant at the 168th amino acid (3A:33109518, C to T, Ala to Val). Likewise, four coding-region variants were identified within *TraesCS3A03G0119800*. Among them, the variant at 3A:33782022 is an in-frame deletion (GGCTCCA to G) that results in the loss of two consecutive amino acids at the protein level, and structural analysis indicates that the two deleted amino acids reside in a membrane-associated cytosolic region (aa 592–593), making this one of the noteworthy putative functional variants in this gene. In contrast, the variants at 3A:33782778, 3A:33782985, and 3A:33783006 are all synonymous substitutions (**Figure 8E**). Although our integrative analyses indicate that *TraesCS3A03G0118200* and *TraesCS3A03G0119800* are currently the most likely candidate genes within this interval, there are still 35 other genes within the interval that also harbored sequence variations (excluding intronic and synonymous mutations) based on WES. The roles of candidate genes in stripe rust resistance still need to be further confirmed through functional validation approaches such as transgenic complementation and gene silencing, thereby supporting a causal relationship with the resistance phenotype.

## 4 Discussion

The stripe rust phenotypes in this study exhibited a clear bimodal distribution, which was partly attributable to the extreme-phenotype selection applied to the population. To improve the efficiency of resistance-locus detection and germplasm identification, we performed extreme-phenotype selection during the initial screening, thereby amplifying the differences between resistant and susceptible groups and producing a more distinct bimodal pattern that increased the power to detect associated loci. This distribution is also consistent with the genetic expectation that resistance is primarily governed by a major-effect locus or an alien introgressed segment: stripe rust resistance of WT78–10 is mainly conferred by the alien introgression, and individuals carrying the *Thinopyrum* translocation diagnostic markers are largely clustered in the low-disease-severity peak, whereas those lacking these markers predominantly fall into the high-disease-severity peak. Meanwhile, some dispersion within each peak suggests that, in addition to the major factor, minor-effect QTLs, genetic background differences, and environmental effects also contribute to resistance; accordingly, another small-effect QTL may exist elsewhere in the genome. Overall, the bimodal distribution is not an abnormality but rather is consistent with the experimental design based on extreme selection and helps enhance the statistical power for resistance-locus dissection and germplasm identification.

Advances in sequencing and genotyping technologies has diversified the platforms available for wheat genetic studies, including SNP genotyping arrays, whole exome sequencing, and whole-genome resequencing. Each approach has distinct strengths and trade-offs in terms of cost, throughput, resolution, and data-processing complexity. For small populations, however, the limited number of available recombination events means that even high-density SNP arrays often detect a large proportion of co-segregating loci, resulting in highly redundant marker information. For example (Cui et al., 2017), reported that in a RIL population of 188 lines genotyped with the wheat 660K array, approximately 96% of polymorphic SNPs were redundant. Therefore, for QTL mapping in small populations, a lower density but well-covered array (e.g., 120K) is usually sufficient for linkage map construction and QTL detection, while also reducing costs and minimizing information redundancy caused by redundant markers. Here, using the wheat 120K SNP array and a RIL population consisting of 70 lines, we constructed a genetic map covering all 21 wheat chromosomes, comprising 578 bin markers with a total length of 2,125.61 cM. Notably, in linkage group 7B, a total of 40,579 markers were assigned, of which 4,878 were polymorphic; however, only 30 non-redundant framework markers were ultimately used for map construction. The 7B linkage group spanned 148.80 cM and exhibited the largest average inter-marker distance (4.96 cM) and the largest gap (27.90 cM) across the entire map. These features indicate pronounced marker redundancy and locally reduced recombination in the 7B linkage group. Based on cytogenetic evidence, this pattern is likely associated with the introgression of alien chromatin in WT78–10 and the accompanying suppression of recombination (Guo et al., 2023). WT78–10 lacks the entire 7B chromosome arm; however, the alien chromosome it carries is homologous to wheat homoeologous group 7, and the introgressed alien segment may, to some extent, partially functionally compensate for the genetic deficit caused by the loss of 7B, allowing some SNP loci to be consistently genotyped and to cluster in linkage analysis. Meanwhile, alien introgression and structural variation are often accompanied by recombination suppression or reduced effective recombination events, which in turn lead to sparse framework markers and enlarged genetic gaps.

A similar pattern was observed in the 6A linkage group. Only 13 non-redundant framework markers were retained on 6A, and the genetic length was markedly shortened (38.30 cM), indicating that effective recombination information available for map construction and QTL localization on this chromosome was extremely limited. This is consistent with previous evidence that alien chromatin almost completely replaced chromosome arm 6AL in WT78-10 (T6AL-Th). Large-scale alien introgression can reduce polymorphism and effective genotyping rates for probes designed from the wheat reference genome and may also suppress homologous pairing and recombination. Consequently, numerous markers become co-segregating, the number of framework markers decreases, and the linkage map becomes compressed, reflecting a severe reduction in effective recombination events (**Supplementary Figure S4**). This greatly diminishes the genetic resolution of the 6A region and represents one of the key reasons why major-effect QTLs (*YrT14*) were difficult to detect on 6A in this study, particularly when the major resistance is primarily derived from the alien segment or from adjacent regions where recombination is suppressed, in which case the mapping difficulty becomes even more pronounced.

A small population size reduces the number of informative recombination events captured, lowers detection power, and inflates effect-size estimates, and also increases the risk of inflated genetic map distances and unstable marker order (Darvasi and Soller, 1997; Doerge, 2002). Therefore, for mapping *QYr.cib-3AS*, we expanded the population to 182 RILs and developed additional markers to further saturate this region, allowing us to narrow the QTL interval and improve mapping resolution. Ultimately, *QYr.cib-3AS* was mapped to the interval between markers dCAPS-78 and dCAPS-83, corresponding to the 29.07–34.33 Mb region on chromosome 3AS in CS v2.1, where no stripe rust–related QTL has been reported to date. Thus far, only one formally designated *Yr* gene, *Yr76*, has been named on chromosome 3AS. *Yr76* originated from the soft white winter wheat cultivar Tyee (Xiang et al., 2016) and was mapped between the flanking markers Xwmc1–Xwmc532, with the locus located near the 1.09 Mb physical position (Wu et al., 2025), and its resistance is no longer effective against the currently prevalent races in China, such as CYR32, CYR33 and CYR34 (Wang et al., 2018; Yao et al., 2019). *QYrsv.swust-3AS* was delimited between markers IWB7237 and IWB8523, with the locus located near 27.19 Mb, its resistance was derived from the durum wheat cultivar Svevo (Zhou et al., 2020a). *QRYr3A.1* was identified at 7.22 Mb and co-segregated with markers Xbarc310 and Xstm844tcac (Lillemo et al., 2008). *Yrq2* was mapped in the Chinese cultivar Xichang 76–9 and located at 22.03 Mb (Cao et al., 2012). *YrHu* was located on the short arm of chromosome 3, between Xcfd79 and XBG604577 (Ma et al., 2016). These loci confer resistance independently, whereas the effect of *QYr.cib-3AS* depends on *YrT14*: when *QYr.cib-3AS* is present, it enhances the effect of *YrT14* by 51.74%. Such synergistic enhancement of disease resistance is not uncommon. For example, the stripe rust resistance locus *Yr84* is conferred by a pair of functionally interdependent and jointly required NLR genes, *CNL* and *NL*, which together elicit a complete and stable resistance response when present simultaneously (Klymiuk et al., 2025). In addition, in wheat powdery mildew resistance, the paired NLR genes *TdCNL1/TdCNL5* have been shown to coordinately regulate resistance. In the susceptible background Fielder, expression of *TdCNL5* alone does not confer resistance to *Blumeria graminis* f. sp. *tritici* (*Bgt*), whereas the simultaneous presence of *TdCNL1* and *TdCNL5* results in stable high-level resistance. The study further indicates that *TdCNL5* acts as a key partner that participates in and enhances *TdCNL1*-mediated immunity (Zhu et al., 2025). Similarly, *PmWR183* encodes adjacent genes *PmWR183-NLR1* and *PmWR183-NLR2*. Functional assays indicate that neither gene alone is sufficient to confer resistance, whereas co-expression restores immunity, and disruption of either gene eliminates resistance (Zhang et al., 2025). Collectively, these examples indicate that plant disease resistance is often determined by multiple closely interacting genetic factors. Some genes/loci may be unable to reconstitute the full resistance phenotype on their own but can act as enhancers/modifiers whose effects are substantially amplified through cooperation with key resistance determinants in specific genetic backgrounds, thereby exhibiting typical background-dependent effects and genetic interactions. It should be noted that *YrT14* confers resistance throughout the entire growth period, whereas whether *QYr.cib-3AS* also functions at the seedling stage remains unclear and requires validation and further dissection in independent seedling inoculation assays. Taken together, considering its origin, effect, resistance type, and physical location, *QYr.cib-3AS* is likely to represent a novel stripe rust resistance locus on 3AS.

Based on CS v2.1, the *QYr.cib-3AS* interval contains 48 predicted genes. Collinearity analysis across 16 chromosome-level wheat genome assemblies showed that the target physical region on chromosome arm 3AS is highly collinear and structurally conserved, with no obvious large-scale insertions, deletions, or translocations. This suggests that the functional variation within this region is more likely attributable to allelic differences, such as SNPs, small InDels, or regulatory-sequence polymorphisms, rather than to structural rearrangements, which is favorable for the identification and cloning of candidate genes for *QYr.cib-3AS*. Based on the transcriptome analysis, only 22 genes displayed detectable expression signals, whereas the remaining genes were either not expressed or expressed at low levels. Among them, four genes showed a significant expression difference between the two parents. Sequence-variation analysis based on WES data revealed variants within *TraesCS3A03G0118200* (Cysteine-rich receptor-like protein kinase 6, CRK) and *TraesCS3A03G0119800* (NRT1/PTR family, NPF) regions.

CRKs represent a major subfamily of RLKs and typically contain extracellular cysteine-rich domains and an intracellular kinase domain; they have been closely associated with abiotic stress responses, plant defense, and programmed cell death (Acharya et al., 2007; Quezada et al., 2019). Previous studies have suggested that CRKs participate in immune and stress signaling, potentially by sensing redox/ROS dynamics in the apoplast and transducing signals to downstream pathways, thereby modulating defense-related responses (Idänheimo et al., 2014). In the study by (Yang et al., 2013), the wheat CRK gene *TaCRK1* was induced upon infection by *Rhizoctonia cerealis*, indicating that CRK-type RLKs may play a role in defense- and stress-associated signal transduction. Notably, silencing *TaCRK1* via virus-induced gene silencing did not obviously impair resistance. *QYr.cib-3AS* confers a resistance feature that is, to some extent, consistent with this pattern. NPF transporters constitute a multifunctional major facilitator superfamily in plants with a broad substrate spectrum. NPF members have been shown to transport a range of immunity-related metabolites and phytohormones, including jasmonates, thereby modulating hormone-mediated defense responses and their systemic signaling. For example (Saito et al., 2015), demonstrated that GTR1/NPF2.10 is a typical jasmonate-responsive transporter closely associated with JA-related transcriptional responses, this may enhance wheat defense against fungal diseases (Yan et al., 2023).

These results suggest that *TraesCS3A03G0118200* and *TraesCS3A03G0119800* are promising candidate genes for *QYr.cib-3AS*. Nevertheless, although the other genes exhibit low expression levels (TPM ≤ 1) or were not expressed in both the resistant and susceptible cultivars, and no sequence differences were detected based on WES, we do not rule out their potential roles in conferring stripe rust resistance.

## 5 Conclusions

In the present study, a QTL associated with resistance to wheat stripe rust was primary mapped to an approximately 5.26 Mb physical interval on chromosome 3A using a combination of BSA-seq and QTL mapping. In addition, (d)CAPS markers suitable for MAS were developed at the *QYr.cib-3AS* locus. *TraesCS3A03G0118200* and *TraesCS3A03G0119800* were proposed as candidate genes underlying this locus. Overall, this study provides practical molecular tools for marker-assisted selection of stripe rust resistance, which will be valuable for maintaining or improving resistance and accelerating the development of superior new cultivars.

## Data Availability

The original contributions presented in the study are included in the article/**Supplementary Material**. Further inquiries can be directed to the corresponding authors.

## Supplementary material

Supplementary Figure 1 Genetic linkage map and collinearity analysis with CS v2.1. (A) Missing data patterns for 70 RIL lines and 578 markers. (B) Genetic linkage map. (C) Collinearity between the genetic map and the reference genome. (D) Physical positions information of markers.

Supplementary Figure 2 Additive effects of *QYr.cib-7AL*. (A) Additive effects of *QYr.cib-7AL* in 2025MA. (B) Additive effects of *QYr.cib-7AL* in 2025XD.

Supplementary Figure 3 Additive effects of *YrT14.*

Supplementary Figure 4 Recombination-based differentiation between the group 6A and *YrT14.*

Supplementary Table 1 Variance components and *H*^2^ for IT in the RIL population and its two parents across environment.

Supplementary Table 2 Significant sliding windows identified by BSA-seq and their ΔSNP-index.

Supplementary Table 3 QTLs for stripe rust resistance detected in the WT78-10 × ZKXM RIL population across three environments and BLUP values.

Supplementary Table 4 Specific markers and primer sequences for *QYr.cib-3AS*. Based on the number of restriction sites within the PCR amplicon, dCAPS-78 was prioritized as the diagnostic marker. In the primer sequences, uppercase letters indicate positions of sequence variants in the pan-genome, while lowercase letters represent conserved positions.

Supplementary Table 5 Two-way ANOVA of the main and interaction effects of *YrT14* and *QYr.cib-3AS.*

Supplementary Table 6 Summary statistics for parental adult-stage leaf transcriptomes.
