# Supplementary material for: The pyramiding of QYr.cib-3AS and YrT14 enhances wheat resistance to stripe rust
Source: Front Plant Sci. 2026 Apr 22;17:1802598. doi: 10.3389/fpls.2026.1802598 (PMC13143962; doi:10.3389/fpls.2026.1802598)
Supplement: Supplementary Figure 1 — Genetic linkage map and collinearity analysis with CS v2.1. (A) Missing data patterns for 70 RIL lines and 578 markers. (B) Genetic linkage map. (C) Collinearity between the genetic map and the reference genome. (D) Physical positions information of markers. [file Supplementaryfile1.docx]

## **Supplementary information**

| 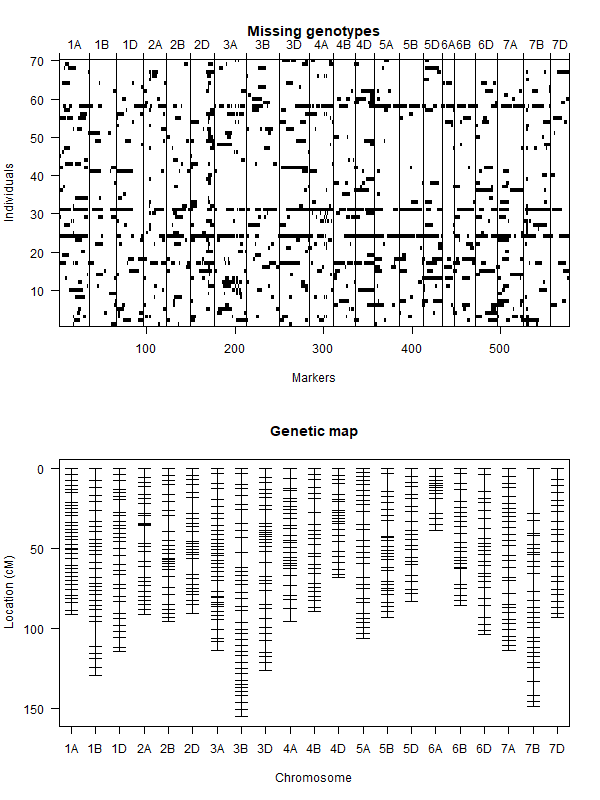 | 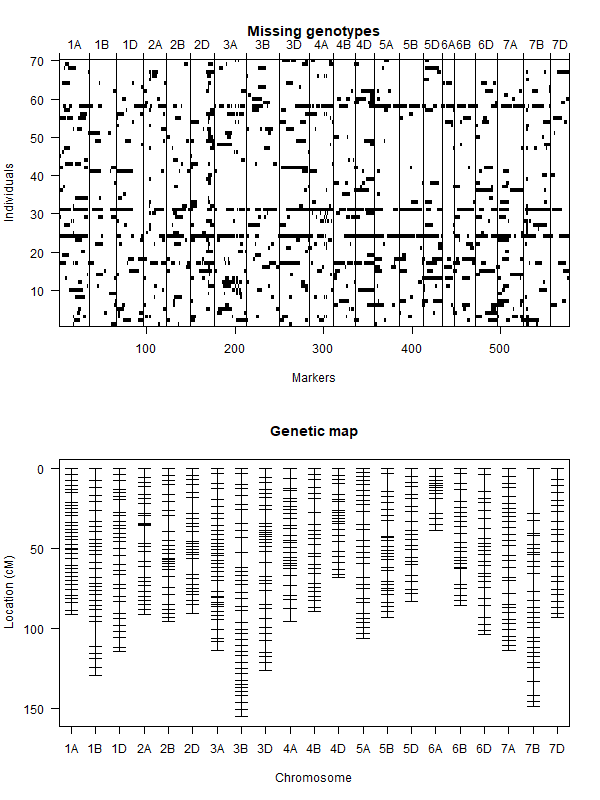 |
| --- | --- |
| A | B |
| 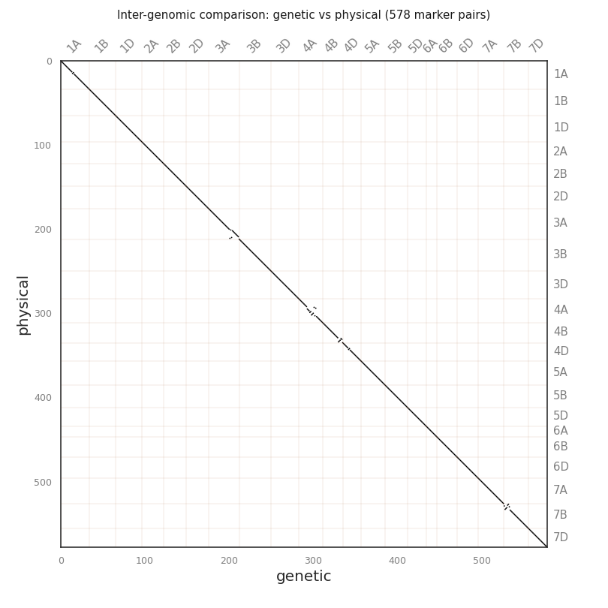 | 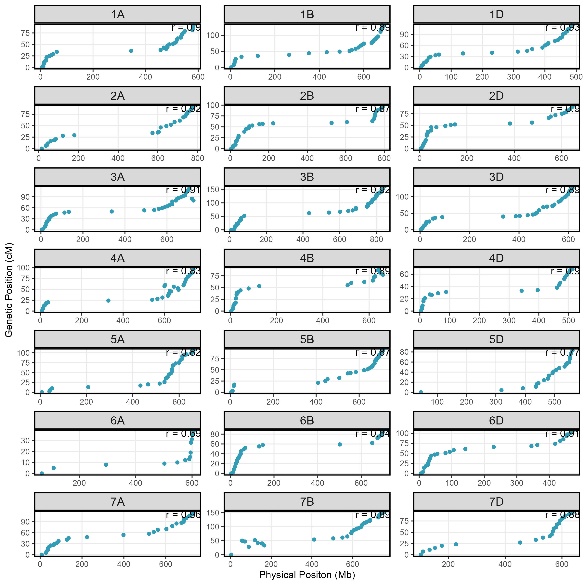 |
| C | D |
| (A) Missing data patterns for 70 RIL lines and 578 markers  (B) Genetic linkage map  (C) Collinearity between the genetic map and the reference genome  (D) Physical positions information of markers | |

Fig. S1 Genetic linkage map and collinearity analysis with CS v2.1
