# Supplementary material for: The pyramiding of QYr.cib-3AS and YrT14 enhances wheat resistance to stripe rust
Source: Front Plant Sci. 2026 Apr 22;17:1802598. doi: 10.3389/fpls.2026.1802598 (PMC13143962; doi:10.3389/fpls.2026.1802598)
Supplement: Supplementary Figure 2 — Additive effects of QYr.cib-7AL. (A) Additive effects of QYr.cib-7AL in 2025MA. (B) Additive effects of QYr.cib-7AL in 2025XD. [file Supplementaryfile2.docx]

## **Supplementary information**

| 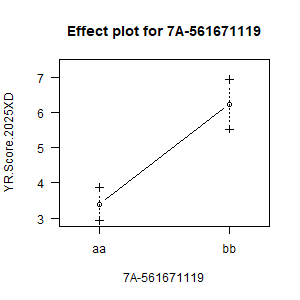 | 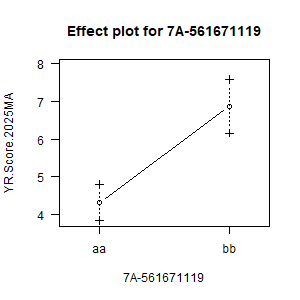 |
| --- | --- |
| A | B |
| (A) Additive effects of *QYr.cib-7AL* in 2025MA  (B) Additive effects of *QYr.cib-7AL* in 2025XD | |

Fig. S2 Additive effects of *QYr.cib-7AL*
