# Supplementary material for: The pyramiding of QYr.cib-3AS and YrT14 enhances wheat resistance to stripe rust
Source: Front Plant Sci. 2026 Apr 22;17:1802598. doi: 10.3389/fpls.2026.1802598 (PMC13143962; doi:10.3389/fpls.2026.1802598)
Supplement: Supplementary Figure 4 — Recombination-based differentiation between the group 6A and YrT14. [file Supplementaryfile4.docx]

## **Supplementary information**

| 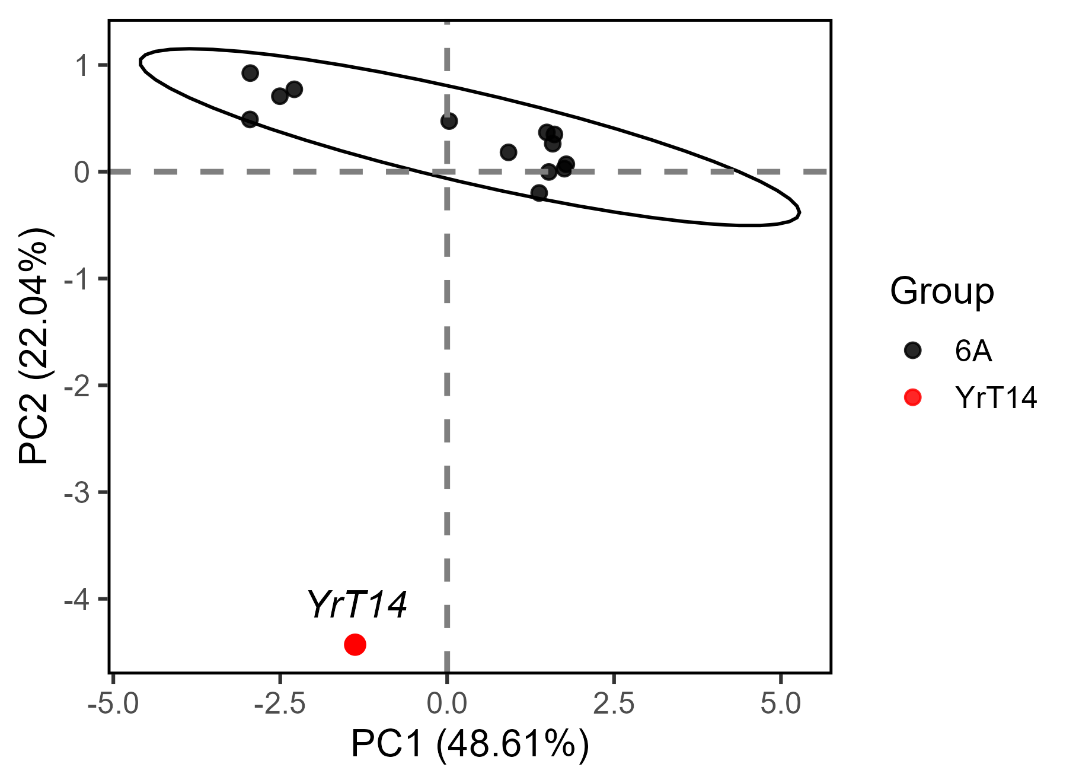 |
| --- |

Fig. S4 Recombination-based differentiation between the group 6A and *YrT14*
