# Supplementary material for: The pyramiding of QYr.cib-3AS and YrT14 enhances wheat resistance to stripe rust
Source: Front Plant Sci. 2026 Apr 22;17:1802598. doi: 10.3389/fpls.2026.1802598 (PMC13143962; doi:10.3389/fpls.2026.1802598)
Supplement: Supplementary Table 1 — Variance components and H2 for IT in the RIL population and its two parents across environment. [file Table1.docx]

## **Supplementary information**

Table S1 Variance components and *H*² for IT in the RIL population and its two parents across environment

| Sources of variation | DF | Mean square | F value |
| --- | --- | --- | --- |
| RILs | 183 | 28.092715 | 12.535 |
| Environments | 2 | 22.191564 | 9.902 |
| Lines × Environments | 366 | 2.241070 | 1.016 |
| Error | 534 | 2.205033 | - |
| *H*^2^ *b* | - | - | 0.9202 |
