# Supplementary material for: The pyramiding of QYr.cib-3AS and YrT14 enhances wheat resistance to stripe rust
Source: Front Plant Sci. 2026 Apr 22;17:1802598. doi: 10.3389/fpls.2026.1802598 (PMC13143962; doi:10.3389/fpls.2026.1802598)
Supplement: Supplementary Table 2 — Significant sliding windows identified by BSA-seq and their ΔSNP-index. [file Table2.docx]

## **Supplementary information**

Table S2 Significant sliding windows identified by BSA-seq and their ΔSNP-index

| Threshold | CHR | Window start (bp) | Window end(bp) | HighB.index | LowB.index | Delta.index | CI95 | CI99 | nSNPs |
| --- | --- | --- | --- | --- | --- | --- | --- | --- | --- |
| P95 | 1B | 1,000,001 | 4,400,000 | 0.57 | 0.13 | 0.44 | ±0.38 | ±0.5 | 17 |
|  | 1B | 8,600,001 | 10,600,000 | 0.59 | 0.25 | 0.34 | ±0.33 | ±0.43 | 16 |
|  | 1B | 47,400,001 | 50,400,000 | 0.61 | 0.94 | -0.32 | ±0.31 | ±0.41 | 11 |
|  | 1D | 39,800,001 | 42,400,000 | 0.31 | 0.66 | -0.35 | ±0.34 | ±0.45 | 30 |
|  | 2B | 800,000,001 | 805,200,000 | 0.36 | 0.73 | -0.37 | ±0.41 | ±0.53 | 28 |
|  | 3A | 24,600,001 | 30,000,000 | 0.58 | 0.19 | 0.39 | ±0.33 | ±0.44 | 22 |
|  | 3B | 32,600,001 | 34,800,000 | 0.43 | 0.85 | -0.41 | ±0.39 | ±0.51 | 14 |
|  | 4A | 562,600,001 | 565,600,000 | 0.77 | 0.41 | 0.36 | ±0.32 | ±0.42 | 12 |
|  | 4B | 20,600,001 | 22,600,000 | 0.43 | 0.85 | -0.41 | ±0.39 | ±0.51 | 14 |
|  | 5A | 582,200,001 | 584,200,000 | 0.46 | 0.12 | 0.34 | ±0.34 | ±0.43 | 34 |
|  | 5B | 388,800,001 | 391,800,000 | 0.49 | 0.12 | 0.38 | ±0.35 | ±0.46 | 16 |
|  | 5B | 684,400,001 | 686,800,000 | 0.27 | 0.66 | -0.39 | ±0.36 | ±0.47 | 22 |
|  | 6B | 143,800,001 | 145,800,000 | 0.34 | 0.69 | -0.35 | ±0.34 | ±0.44 | 11 |
|  | 6B | 283,600,001 | 286,000,000 | 0.29 | 0.69 | -0.40 | ±0.37 | ±0.48 | 14 |
|  | 6B | 722,200,001 | 724,400,000 | 0.56 | 0.98 | -0.42 | ±0.41 | ±0.53 | 11 |
|  | 7A | 272,400,001 | 274,400,000 | 0.55 | 0.14 | 0.41 | ±0.38 | ±0.5 | 10 |
|  | 7B | 6,800,001 | 18,000,000 | 0.51 | 0.89 | -0.38 | ±0.36 | ±0.46 | 13 |
| P99 | 3A | 27,600,001 | 29,600,000 | 0.60 | 0.18 | 0.42 | ±0.32 | ±0.42 | 14 |
