# Supplementary material for: The pyramiding of QYr.cib-3AS and YrT14 enhances wheat resistance to stripe rust
Source: Front Plant Sci. 2026 Apr 22;17:1802598. doi: 10.3389/fpls.2026.1802598 (PMC13143962; doi:10.3389/fpls.2026.1802598)
Supplement: Supplementary Table 3 — QTLs for stripe rust resistance detected in the WT78-10 × ZKXM RIL population across three environments and BLUP values. [file Table3.docx]

## **Supplementary information**

Table S3 QTLs for stripe rust resistance detected in the WT78-10 × ZKXM RIL population across three environments and BLUP values

| QTL | Chr | Env | Left marker | Right marker | Genetic position | LOD | PVE |
| --- | --- | --- | --- | --- | --- | --- | --- |
| *QYr.cib-3AS* | 3A | 2024XD | 3A-26431044 | 3A-29976997 | 20 | 2.57 | 20.10 |
|  |  | 2025XD | 3A-26431044 | 3A-29976997 | 20 | 3.24 | 25.21 |
|  |  | 2025MA | 3A-29976997 | 3A-37517932 | 25 | 3.58 | 21.45 |
|  |  | BLUP | 3A-26431044 | 3A-29976997 | 20 | 3.06 | 19.13 |
| *QYr.cib-7AL* | 7A | 2025XD | 7A-543126780 | 7A-561671119 | 65 | 2.96 | 19.03 |
|  |  | 2025MA | 7A-543126780 | 7A-561671119 | 65 | 3.07 | 16.53 |
