# Supplementary material for: The pyramiding of QYr.cib-3AS and YrT14 enhances wheat resistance to stripe rust
Source: Front Plant Sci. 2026 Apr 22;17:1802598. doi: 10.3389/fpls.2026.1802598 (PMC13143962; doi:10.3389/fpls.2026.1802598)
Supplement: Supplementary Table 4 — Specific markers and primer sequences for QYr.cib-3AS. Based on the number of restriction sites within the PCR amplicon, dCAPS-78 was prioritized as the diagnostic marker. In the primer sequences, uppercase letters indicate positions of sequence variants in the pan-genome, while lowercase letters represent conserved positions. [file Table4.docx]

## **Supplementary information**

Table S4 Specific markers and primer sequences for *QYr.cib-3AS*

| Marker | Sequence | Enzyme | Digest time |
| --- | --- | --- | --- |
| dCAPS-78-F | tatcccgtcctatcgtcttcca | BamHI | 3h |
| dCAPS-78-R | actctgttgatgtgctggagtt |  |  |
| dCAPS-56-F | tccatAgtaactgacaacTgctTc | TaqαI | 3h |
| dCAPS-56-R | agtaatctTctgttgTcccggC |  |  |

Note: Based on the number of restriction sites within the PCR amplicon, dCAPS-78 was prioritized as the diagnostic marker. In the primer sequences, uppercase letters indicate positions of sequence variants in the pan-genome, while lowercase letters represent conserved positions.
