# Supplementary material for: The pyramiding of QYr.cib-3AS and YrT14 enhances wheat resistance to stripe rust
Source: Front Plant Sci. 2026 Apr 22;17:1802598. doi: 10.3389/fpls.2026.1802598 (PMC13143962; doi:10.3389/fpls.2026.1802598)
Supplement: Supplementary Table 5 — Two-way ANOVA of the main and interaction effects of YrT14 and QYr.cib-3AS. [file Table5.docx]

## **Supplementary information**

Table S5 Two-way ANOVA of the main and interaction effects of *YrT14* and *QYr.cib-3AS*

| Sources | Sum Sq | DF | F value | Pr (>F) |  |
| --- | --- | --- | --- | --- | --- |
| *YrT14* | 610.14 | 1 | 213.445 | < 2.20E-16 | *** |
| *QYr.cib-3AS* | 29.65 | 1 | 10.373 | 0.001553 | ** |
| *YrT14*: *QYr.cib-3AS* | 19.76 | 1 | 6.9142 | 0.009397 | ** |
