# Supplementary material for: The pyramiding of QYr.cib-3AS and YrT14 enhances wheat resistance to stripe rust
Source: Front Plant Sci. 2026 Apr 22;17:1802598. doi: 10.3389/fpls.2026.1802598 (PMC13143962; doi:10.3389/fpls.2026.1802598)
Supplement: Supplementary Table 6 — Summary statistics for parental adult-stage leaf transcriptomes. [file Table6.docx]

## **Supplementary information**

Table S6 Summary statistics for parental adult-stage leaf transcriptomes

| Name | Clean reads(M) | Clean bases(G) | Q20 (%) | Q30 (%) | GC percent (%) |
| --- | --- | --- | --- | --- | --- |
| WT78-10-R1 | 83.047286 | 12.375153 | 97.15% | 92.37% | 53.51% |
| WT78-10-R2 | 81.76914 | 12.160786 | 97.47% | 93.28% | 53.52% |
| WT78-10-R3 | 90.776774 | 13.544396 | 97.60% | 93.49% | 53.16% |
| ZKXM-S1 | 64.884018 | 9.703271 | 97.22% | 92.42% | 50.77% |
| ZKXM-S2 | 91.123668 | 13.601147 | 97.57% | 93.33% | 49.82% |
| ZKXM-S3 | 76.02807 | 11.339978 | 97.53% | 93.26% | 49.38% |
| WT78-10-R under field infection with a mixture of stripe rust strains | | | | | |
| ZKXM-S under field infection with a mixture of stripe rust strains | | | | | |
| 1, 2, 3: biological replicate | | | | | |
